# Adaptive suspension state estimation based on IMMAKF on variable vehicle speed, road roughness grade and sprung mass condition

**DOI:** 10.1038/s41598-023-49766-y

**Published:** 2024-01-19

**Authors:** Xiao Wu, Wenku Shi, Hong Zhang, Zhiyong Chen

**Affiliations:** 1 State Key Laboratory of Automotive Simulation and Control, Jilin University, Changchun, China; 2 Weifang Economic School, Zhucheng, China

**Keywords:** Mechanical engineering, Information technology

## Abstract

Vehicle speed, road roughness grade and sprung mass are the three main factors to influence suspension control and state estimation. Aiming at the problem that fixed state observer cannot guarantee the estimation accuracy of suspension with driving scenario changes, a suspension state observer based on interactive multiple model adaptive Kalman filter (IMMAKF) is established. Firstly, an adaptive control suspension is proposed based on LQR algorithm and multi-objective optimization algorithm, which can automatically adjust the controller parameters according to the vehicle speed, road roughness grade and sprung acceleration parameters, so as to keep the optimal control effect of the suspension. Secondly, the theoretical model of IMMAKF is derived, and two kinds of IMMAKF suspension state observers and controllers are established. Finally, a simulation condition with the vehicle speed, road roughness grade and sprung mass changing simultaneously is established. The simulation results shows that: compared with ordinary IMMKF, AKF and KF observers, the estimation accuracy of IMMAKF5 is improved. Except for state observation, IMMAKF can be used to identify the road roughness grade and estimate the suspension sprung mass.

## Introduction

Suspension is a significant system for vehicles to alleviate road excitation. Active suspension systems use controlled actuator to improve ride comfort and road holding stability^1^. During the control process, the suspension controller needs to get the precise state data through state observer to calculate the optimal controller parameters. The design of controller and state observer is the core of suspension control^2,3^.

Ordinary active suspension systems mostly regard state equations as fixed values which are designed by researchers according to the most used working conditions^4^. When the vehicle is driving on special working conditions, the vibration performance like ride comfort is often poor^5,6^. Based on this problem, the adaptive suspension control strategy is proposed^7–9^. Adaptive suspensions can change state equations according to working conditions to optimize the vibration performance. Vehicle longitudinal speed, road roughness grade and sprung mass are the three main factors that affect the vibration performance of suspensions^10^. The calculation of these three factors has a significant impact on the control effect of the suspension. The identification of vehicle longitudinal speed can be divided into direct estimation^11,12^ and indirect estimation^13,14^. At the present, with the development of sensors, the use of high-precision onboard speed sensor enables the vehicle to calculate longitudinal speed directly. The estimation of road roughness grade can be divided into two types: vision sensors based measurement^15–17^ and state observation based measurement^18–20^. The second method has high accuracy, low cost and good real-time performance. For vehicles, since the total weight of passengers and cargo is random, the value of sprung mass is difficult to calculate. Therefore, the sprung mass is set as a fixed parameter by most current studies^10^ or a variable parameter within a certain range. For suspensions with variable sprung mass, the sprung mass is usually set as a state parameter, and the value of sprung mass is estimated by state observation, which is complicated for modeling. Based on this problem, a new suspension controller is proposed to maintain the optimal vibration state of the suspension without calculating the exact value of the sprung mass.

The state equations of adaptive suspensions are changed with the change of working conditions (such as vehicle speed, road roughness grade and sprung mass). If state observers of adaptive suspensions are fixed, the precision of state estimation will be low^21,22^. Therefore, fixed state observers are not suitable for the state estimation of adaptive suspensions. The Kalman filter (KF) has high estimation accuracy and low cost, which has been commonly used as observers for vehicle control^23–25^ and suspension control^26–30^. In recent years, variable state KF observers are proposed and applied for state estimation of adaptive suspensions, like adaptive Kalman filter (AKF) and interactive multiple model Kalman filter (IMMKF). AKF observer can follow the state of the suspension controller to improve the estimation accuracy. In Ref.^31^, suspension sprung acceleration is utilized to identify road roughness grade and determine state equations, then the corresponding AKF is used to estimate the system. In Ref.^32^, an adaptive unscented Kalman filter (AUKF) observer is proposed and product-based neural network (PNN) is utilized for road classification. In Ref.^33^, a multi-mode switching control strategy of an intelligent suspension system is proposed under different road conditions and the road input is estimated by AKF. IMMKF uses multiple models in parallel for state estimation to reduce observation errors. In Ref.^34^, the variable state and road roughness grade are estimated by an interactive multi-model untracked Kalman filter (IMMUKF) observer. The working conditions of the above studies are changing road roughness grade. However, in the actual situation, for adaptive suspensions, the vehicle speed, road roughness grade and sprung mass all have great impacts on system control and state estimation, so just changing the road is not enough to comprehensively improve the suspension performance. For more complex working conditions, AKF and IMMKF have some disadvantages. For AKF, accurate working conditions like road roughness grade and sprung mass should be calculated to switch the observer mode, which is a complicated process. For IMMKF, the number of sub-models increases with the increase of working conditions. If the number of sub-models is too large, the number of parallel computing models is large, which occupies more computer memory and makes program harder.

Based on the idea of changing vehicle speed, road roughness grade and sprung mass simultaneously, IMMAKF observer is proposed for state observation of adaptive suspension. The main contributions of this paper are:

- The IMMAKF suspension state estimation theory is proposed. IMMAKF observer can improve the state estimation accuracy under changing vehicle speed, road grade and sprung mass.
- A new sprung mass estimation theory is proposed. The sprung mass is estimated by model interaction probabilities of IMMAKF without taking it as one of the state parameters to calculate.
- A new adaptive suspension controller system is proposed. The controller system can improve the ride comfort on variable working conditions.

The rest of this paper is organized as follows. Section "Adaptive control suspension model" introduces a new adaptive suspension controller model; Section "Suspension IMMAKF State Estimation Theory" presents the theory of IMMAKF state estimation; Section "Suspension state estimation and control based on IMMAKF" details simulation and comparison and Section "Conclusions" concludes this paper.

## Adaptive control suspension model

In this section, an adaptive control suspension model is established based on LQG and fuzzy control theory to make the vehicle in the optimal state when driving on different working conditions (road roughness grade, vehicle velocity and sprung mass).

### LQR Half-car suspension control system modeling

In this part, the half-car suspension system is presented, and the LQR suspension control theory is discussed.

**Figure 1:**
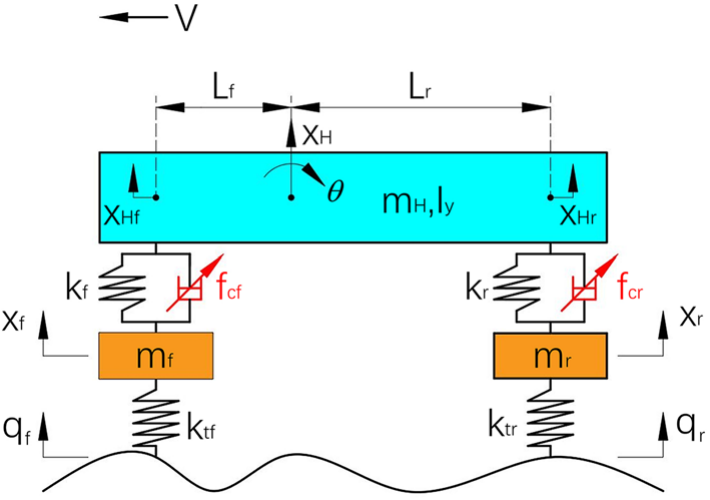
The half-car suspension model.

The half-car suspension model is established as Fig. 1. The suspension consists of a spring and an actuator at the front and rear part respectively. The tire is modeled as a linear spring.

Where,$m_{H}$ denotes the sprung mass;$I_{y}$ is the pitch inertia;$m_{f}$ and $m_{r}$ are front and rear unsprung mass, respectively;$k_{f}$,$k_{r}$ represent front and rear suspension stiffness, respectively;$k_{tf}$,$k_{tr}$ indicate front and rear tire stiffness, respectively;$x_{H}$ is the sprung mass vertical displacement at the Center of Gravity(CG) point;$\theta$ is pitch angle of the sprung mass at the CG point;$x_{Hf}$ and $x_{Hr}$ are the vertical displacement of front and rear sprung mass, respectively;$x_{f}$ and $x_{r}$ are the vertical displacement of front and rear unsprung mass, respectively;$q_{f}$ and $q_{r}$ are the road input at front and rear tire, respectively;$f_{cf}$ and $f_{cr}$ are controlled actuator force at front and rear suspension, respectively; *V* is the longitudinal vehicle speed.

Parameters of the suspension model are shown in Table 1:

**Table 1:** Parameters of the half-car suspension model.

Symbol	Meaning	Unit	Value
$m_{H}$	Sprung mass	kg	300 ~ 500
$I_{y}$	Moment of inertia	${\text{kg m}}^{{2}}$	1200 ~ 1500
$m_{f}$	Front unsprung mass	kg	30
$m_{r}$	Rear unsprung mass	kg	30
$k_{f}$	Front suspension spring stiffness	${\text{N m}}^{ - 1}$	15,000
$k_{r}$	Rear suspension spring stiffness	${\text{N m}}^{ - 1}$	15,000
$k_{tf}$	Front tire stiffness	${\text{N m}}^{ - 1}$	150,000
$k_{tr}$	Rear tire stiffness	${\text{N m}}^{ - 1}$	150,000
$L_{f}$	Distance between mass center to front wheel	m	1.187
$L_{r}$	Distance between mass center to front wheel	m	1.533
*V*	Vehicle velocity	${\text{N m}}^{ - 1}$	10 ~ 30

Vertical dynamics equations for the half-car suspension model shown in Fig. 1 can be expressed as:

1 $$ \begin{gathered} m_{f} \ddot{x}_{f} + k_{tf} (x_{f} - q_{f} ) + F_{f} = 0 \hfill \\ m_{r} \ddot{x}_{r} + k_{tr} (x_{r} - q_{r} ) + F_{r} = 0 \hfill \\ m_{H} \ddot{x}_{H} - F_{f} - F_{r} = 0 \hfill \\ I_{y} \ddot{\theta } + L_{f} F_{f} - L_{r} F_{r} = 0 \hfill \\ \end{gathered} $$

where,

2 $$ \begin{gathered} F_{f} = f_{cf} + k_{f} (x_{f} - x_{Hf} ) \hfill \\ F_{r} = f_{cr} + k_{r} (x_{r} - x_{Hr} ) \hfill \\ \end{gathered} $$

For the suspension model, and can be approximately calculated as

3 $$ \begin{gathered} x_{Hf} \approx x_{H} + L_{f} \theta ;\ddot{x}_{Hf} \approx \ddot{x}_{H} + L_{f} \ddot{\theta } \hfill \\ x_{Hr} \approx x_{H} - L_{r} \theta ;\ddot{x}_{Hr} \approx \ddot{x}_{H} - L_{r} \ddot{\theta } \hfill \\ \end{gathered} $$

Equation (1) can be rewritten as

4 $$ {\mathbf{A}}{\ddot{\mathbf{x}}} + {\mathbf{Bx}} + {\mathbf{Cq}} + {\mathbf{Df}}_{{\mathbf{c}}} = {\mathbf{0}}_{4 \times 1} $$

where,

$${\mathbf{q}} = \left[ {\begin{array}{*{20}c} {q_{f} } \\ {q_{r} } \\ \end{array} } \right]$$; $${\mathbf{f}}_{{\mathbf{c}}} = \left[ {\begin{array}{*{20}c} {f_{cf} } \\ {f_{cr} } \\ \end{array} } \right]$$; $${\mathbf{A}} = \left[ {\begin{array}{*{20}c} {m_{H} } & 0 & 0 & 0 \\ 0 & {I_{y} } & 0 & 0 \\ 0 & 0 & {m_{f} } & 0 \\ 0 & 0 & 0 & {m_{r} } \\ \end{array} } \right]$$; $${\mathbf{C}} = \left[ {\begin{array}{*{20}c} {\begin{array}{*{20}c} 0 \\ 0 \\ { - k_{tf} } \\ 0 \\ \end{array} } & {\begin{array}{*{20}c} 0 \\ 0 \\ 0 \\ { - k_{tr} } \\ \end{array} } \\ \end{array} } \right]$$; $${\mathbf{B}} = \left[ {\begin{array}{*{20}c} {k_{f} + k_{r} } & {k_{f} L_{f} - k_{r} L_{r} } & { - k_{f} } & { - k_{r} } \\ {k_{r} L_{r} - k_{f} L_{f} } & { - k_{f} L_{f}^{2} - k_{r} L_{r}^{2} } & {k_{f} L_{f} } & { - k_{r} L_{r} } \\ { - k_{f} } & { - k_{f} L_{f} } & {k_{tf} + k_{f} } & 0 \\ { - k_{r} } & {k_{r} L_{r} } & 0 & {k_{tr} + k_{r} } \\ \end{array} } \right]$$; $${\mathbf{D}} = \left[ {\begin{array}{*{20}c} {\begin{array}{*{20}c} { - 1} \\ {L_{f} } \\ 1 \\ 0 \\ \end{array} } & {\begin{array}{*{20}c} { - 1} \\ { - L_{r} } \\ 0 \\ 1 \\ \end{array} } \\ \end{array} } \right]$$ $${\mathbf{x}} = \left[ {\begin{array}{*{20}c} {x_{H} } \\ \theta \\ {x_{f} } \\ {x_{r} } \\ \end{array} } \right]$$.

According to^29^, $q_{f}$ and $q_{r}$ can be calculated as

5 $$ \begin{gathered} \dot{q}_{f} = - 2\pi f_{0} q_{f} + 2\pi n_{0} \sqrt {G_{q} (n_{0} )V} \omega_{f} \hfill \\ \dot{q}_{r} = - 2\pi f_{0} q_{r} + 2\pi n_{0} \sqrt {G_{q} (n_{0} )V} \omega_{r} \hfill \\ \end{gathered} $$

where, $G_{q} (n_{0} )$ is the power spectral density (PSD) of the road profile^35^. $f_{0}$ is the lowest cutoff frequency, and $f_{0} = 0.01{\text{Hz}}$^36^. $n_{0}$ is the reference spatial frequency, and $n_{0} = 0.1{\text{m}}^{ - 1}$^37^. $\omega_{f}$ and $\omega_{r}$ denotes zero-mean band-limited white noise.

The road profile is homogeneous and isotropic Gaussian process. The function of road profile^37^ is shown as Eq. (6).

6 $$ G_{q} (n_{k} ) = G_{q} (n_{0} )\left( {\frac{{n_{k} }}{{n_{0} }}} \right)^{ - W} $$

where, $n_{k}$ is the spatial frequency, *W* is the road reference coefficient, $W = 2$.

According to ISO 8608^38^, the road roughness grades are defined from level A to D by $G_{q} (n_{0} )( \times 10^{ - 6} {\text{m}}^{3} )$. The range of A grade is [0,32], B is [32,128], C is [128,512] and D is [512,2048]. The road grade corresponding to the geometric mean value of $G_{q} (n_{0} )$ in the interval is called ISO standard road. The value of ISO A is 16, ISO B is 64, ISO C is 256 and ISO D is 1024. The ISO standard road is used to as road model in Section "Suspension state estimation and control based on IMMAKF".

According to ISO 8608^38^, the road grade index *d* is defined as:

7 $$ d = \log_{2} \left[ {G_{q} \left( {n_{0} } \right) \times 10^{6} } \right] $$

According to Eq. (7), the road grade is simplified by *d*. The *d* of ISO A is 4, ISO B is 6, ISO C is 8, ISO D is 10.

According to Eq. (4) and (5), the state space equations of the half-car suspension system is expressed as Eq. (8).

8 $$ \left[ {\begin{array}{*{20}c} {\ddot{\mathbf{x}}} \\ {{\dot{\mathbf{x}}}} \\ {{\dot{\mathbf{q}}}} \\ \end{array} } \right]{\mathbf{ = }}\left[ {\begin{array}{*{20}c} {{\mathbf{0}}_{{{\mathbf{4*4}}}} } & {{\mathbf{ - A}}^{{{\mathbf{ - 1}}}} {\mathbf{B}}} & {{\mathbf{ - A}}^{{{\mathbf{ - 1}}}} {\mathbf{C}}} \\ {{\mathbf{I}}_{{{\mathbf{4*4}}}} } & {{\mathbf{0}}_{{{\mathbf{4*4}}}} } & {{\mathbf{0}}_{{{\mathbf{4*2}}}} } \\ {{\mathbf{0}}_{{{\mathbf{2*4}}}} } & {{\mathbf{0}}_{{{\mathbf{2*4}}}} } & {{\text{M}}_{{{\mathbf{2*2}}}} } \\ \end{array} } \right]\left[ {\begin{array}{*{20}c} {{\dot{\mathbf{x}}}} \\ {\mathbf{x}} \\ {\mathbf{q}} \\ \end{array} } \right]{\mathbf{ + }}\left[ {\begin{array}{*{20}c} {{\mathbf{ - A}}^{{{\mathbf{ - 1}}}} {\mathbf{D}}} \\ {{\mathbf{0}}_{{{\mathbf{4*2}}}} } \\ {{\mathbf{0}}_{{{\mathbf{2*2}}}} } \\ \end{array} } \right]\left[ {\begin{array}{*{20}c} {{\mathbf{f}}_{{{\mathbf{cf}}}} } \\ {{\mathbf{f}}_{{{\mathbf{cr}}}} } \\ \end{array} } \right]{\mathbf{ + }}\left[ {\begin{array}{*{20}c} {{0}_{{8*2}} } \\ {{\text{N}}_{{2*2}} } \\ \end{array} } \right]\left[ {\begin{array}{*{20}c} {\omega_{{\mathbf{f}}} } \\ {\omega_{{\mathbf{r}}} } \\ \end{array} } \right] $$

Equation (8) is rewritten as

9 $$ \begin{gathered} {\dot{\text{X}}} = {\text{A}}_{0} {\text{X}} + {\text{B}}_{0} {\text{u}} + {\text{G}}_{0} {\upomega } \hfill \\ {\text{Y}} = {\text{C}}_{0} {\text{X}} + {\text{D}}_{0} {\text{u}} \hfill \\ \end{gathered} $$

where,

$$ {\mathbf{X = }}\left[ {\begin{array}{*{20}c} {{\dot{\mathbf{x}}}} \\ {\mathbf{x}} \\ {\mathbf{q}} \\ \end{array} } \right];{\mathbf{u = f}}_{{\mathbf{c}}} {\mathbf{ = }}\left[ {\begin{array}{*{20}c} {{\mathbf{f}}_{{{\mathbf{cf}}}} } \\ {{\mathbf{f}}_{{{\mathbf{cr}}}} } \\ \end{array} } \right];{\mathbf{\omega = }}\left[ {\begin{array}{*{20}c} {\omega_{{\mathbf{f}}} } \\ {\omega_{{\mathbf{r}}} } \\ \end{array} } \right];{\mathbf{G}}_{{\mathbf{0}}} {\mathbf{ = }}\left[ {\begin{array}{*{20}c} {{0}_{{8*2}} } \\ {{\text{N}}_{{8*2}} } \\ \end{array} } \right];{\text{q}} = \left[ {\begin{array}{*{20}c} {q_{f} } \\ {q_{r} } \\ \end{array} } \right];{\text{M}} = \left[ {\begin{array}{*{20}c} { - 2\pi f_{0} } & 0 \\ 0 & { - 2\pi f_{0} } \\ \end{array} } \right] $$

$$ {\mathbf{A}}_{{\mathbf{0}}} {\mathbf{ = }}\left[ {\begin{array}{*{20}c} {{\mathbf{0}}_{{{\mathbf{4*4}}}} } & {{\mathbf{ - A}}^{{{\mathbf{ - 1}}}} {\mathbf{B}}} & {{\mathbf{ - A}}^{{{\mathbf{ - 1}}}} {\mathbf{C}}} \\ {{\mathbf{I}}_{{{\mathbf{4*4}}}} } & {{\mathbf{0}}_{{{\mathbf{4*4}}}} } & {{\mathbf{0}}_{{{\mathbf{4*2}}}} } \\ {{\mathbf{0}}_{{{\mathbf{2*4}}}} } & {{\mathbf{0}}_{{{\mathbf{2*4}}}} } & {{\text{M}}_{{{\mathbf{2*2}}}} } \\ \end{array} } \right];{\mathbf{B}}_{{\mathbf{0}}} {\mathbf{ = }}\left[ {\begin{array}{*{20}c} {{\mathbf{ - A}}^{{{\mathbf{ - 1}}}} {\mathbf{D}}} \\ {{\mathbf{0}}_{{{\mathbf{4*2}}}} } \\ {{\mathbf{0}}_{{{\mathbf{2*2}}}} } \\ \end{array} } \right];{\text{N}} = \left[ {\begin{array}{*{20}c} {2\pi \sqrt {G_{q} (n_{0} )V} } & 0 \\ 0 & {2\pi \sqrt {G_{q} (n_{0} )V} } \\ \end{array} } \right];{\text{Y}} = \left[ {\begin{array}{*{20}c} {\begin{array}{*{20}c} {\ddot{x}_{H} } & {\ddot{\theta }} & {x_{Hf} - x_{f} } \\ \end{array} } & {\begin{array}{*{20}c} {x_{Hr} - x_{r} } & {x_{f} - q_{f} } & {x_{r} - q_{r} } \\ \end{array} } \\ \end{array} } \right]^{\prime} $$

To balance the ride comfort and handling stability of the vehicle under variable working conditions, the optimal controllable actuator force $f_{cf}$ and $f_{cr}$ are calculated by LQR algorithm.

The LQR controller is designed as Eq. (10):

10 $$ J = \frac{1}{2}\int_{0}^{\infty } {J_{0} dt} = \frac{1}{2}\int_{0}^{\infty } {\left( {X^{T} QX + U^{T} RU + 2X^{T} NU} \right)} dt $$

where $J_{0}$ is the suspension comprehensive performance index.$J_{0}$ is calculated as Eq. (11).

11 $$  \begin{gathered}   J_{0}  = q_{1} (x_{{Hf}}  - x_{f} )^{2}  + q_{2} (\ddot{x}_{{Hf}} )^{2}  + q_{3} (x_{f}  - q_{f} )^{2}  \hfill \\   \quad \; + q_{4} (x_{{Hr}}  - x_{r} )^{2}  + q_{5} (\ddot{x}_{{Hr}} )^{2}  + q_{6} (x_{r}  - q_{r} )^{2}  \hfill \\  \end{gathered}   $$

According to Eq. (3), $J_{0}$ is calculated as:

12 $$ \begin{gathered}   J_{0}  = q_{1} (x_{H}  + L_{f} \theta  - x_{f} )^{2}  + q_{2} (\ddot{x}_{H}  + L_{f} \ddot{\theta })^{2}  + q_{3} (x_{f}  - q_{f} )^{2}  \hfill \\   \quad \; + q_{4} (x_{H}  - L_{r} \theta  - x_{r} )^{2}  + q_{5} (\ddot{x}_{H}  - L_{r} \ddot{\theta })^{2}  + q_{6} (x_{r}  - q_{r} )^{2}  \hfill \\  \end{gathered}   $$

where, $q_{1}$ and $q_{4}$ are the controller parameters of suspension deflection of front and rear suspension respectively, $q_{2}$ and $q_{5}$ are the controller parameters of sprung mass acceleration of front and rear suspension respectively, $q_{3}$ and $q_{6}$ are the controller parameters of tire deflection of front and rear tire respectively.

The optimal controllable actuator force $f_{cf}$ and $f_{cr}$ are calculated by Eq. (13)

13 $$ f_{c} = - Kx = - (B^{T} P + N^{T} )x $$

where, *P* is the solution of Riccati equation^20^:

14 $$ PA + A^{T} P - (PB + N)R^{ - 1} (B^{T} P + N^{T} ) + q = 0 $$

When the best controllable actuator force is solved by Eq. (13), the optimal state space equations of LQR half-car suspension system are

15 $$ \begin{gathered} \dot{x} = A_{LQG} x + B_{LQG} \omega \hfill \\ y = C_{LQG} x \hfill \\ \end{gathered} $$

where,

$$ \begin{gathered} A_{LQG} = A - BK \hfill \\ B_{LQG} = G \hfill \\ C_{LQG} = C - EK \hfill \\ \end{gathered} $$

In the actual control process, according to Eq. (15), the system state equations is discretized as Eq. (16).

16 $$ \begin{gathered} x(k + 1) = G_{LQG} x(k) + H_{LQG} \omega (k) \hfill \\ y(k) = C_{LQG} x(k) + v(k) \hfill \\ \end{gathered} $$

where,* k* is the sampling time, $\omega (k)$ is the process noise, and $\nu (k)$ is the sampling noise. $\omega (k)$ and $\nu (k)$ are Gaussian sequences with mean value of 0, and the time interval is 0.01 s.

### Multi-objective optimization of suspension controller parameters

In this part, the controller parameters of LQR algorithm ($q_{1}$ to $q_{6}$) under variable working conditions are optimized by the second non-dominated sorting genetic algorithm (NSGA-II).

For suspension optimal control, the optimization of the suspension controller parameters is regarded as a multi-objective optimization problem (MOOP). The road handling and ride comfort are two conflict properties. It's arduous to get a satisfactory ride comfort without sacrificing the control ability and vice versa. On the other hand, for different working conditions, the optimal LQR controller parameters are different. To keep the best suspension vibration effect under variable working conditions, LQR suspension controller parameters ($q_{1}$ to $q_{6}$) are optimized by the NSGA-II algorithm. In order to facilitate the optimization process, set $q_{1} = q_{4}$, $q_{2} = q_{5}$,$q_{3} = q_{6}$.

Since $q_{3}$ and $q_{6}$ have little influence on the driving characteristics of suspension, set $q_{3} = q_{6} = 0.0001$. The optimization process is a MOOP. The optimization decision variables are defined as $q_{1} (q_{4} )$ and $q_{2} (q_{5} )$. The objective functions, optimization goals and constraints are shown as Eq. (17).

17 $$ \begin{gathered} \min \left| {\ddot{x}_{H} } \right|(RMS) \hfill \\ \min 0.5(\max (\left| {x_{Hf} - x_{f} } \right|) + \max (\left| {x_{Hr} - x_{r} } \right|)) \hfill \\ S.T.\max (\left| {x_{Hf} - x_{f} } \right|) < 0.15 \hfill \\ S.T.\max (\left| {x_{Hr} - x_{r} } \right|) < 0.15 \hfill \\ S.T.0 \le q_{1} \le 10^{9} \hfill \\ S.T.0 \le q_{2} \le 2000 \hfill \\ \end{gathered} $$

For NSGA-II optimization, the population size is 30, the number of generations is 100. The vehicle speed is 10 ~ 30 ${\text{m s}}^{{ - 1}}$(10, 15, 20, 25, 30 ${\text{m s}}^{{ - 1}}$), road roughness grade is A ~ D class (A, B, C and D) and sprung mass is 300 ~ 500 kg (300, 350, 400, 450, 500 kg). With the combination of these conditions, 100 kinds of different conditions are obtained and optimized by NSGA-II.

**Figure 2:**
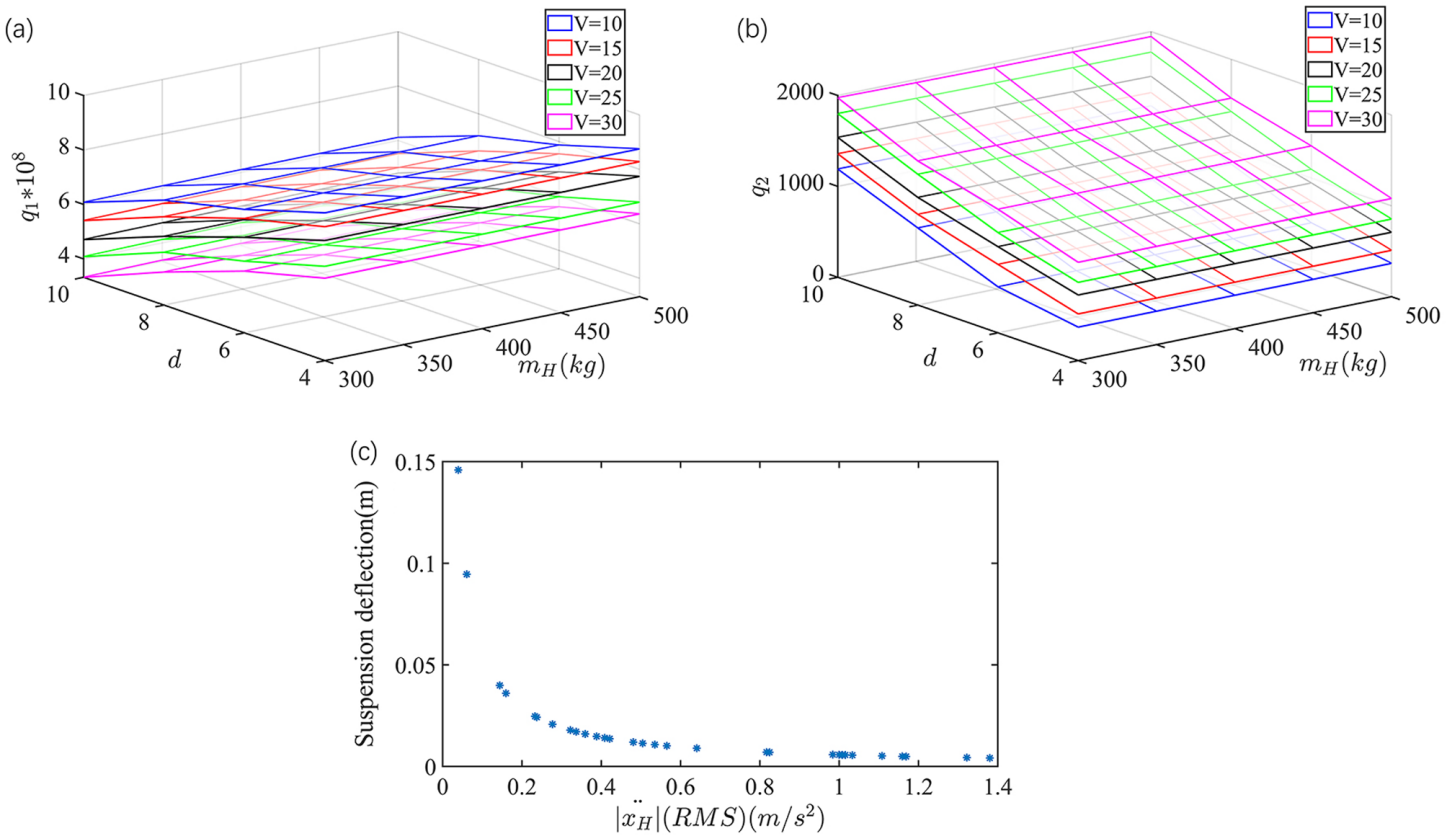
Optimization result of q1 and q2.

The optimization results of vehicle speed 20 ${\text{m s}}^{{ - 1}}$, road grade C and sprung mass 400 kg are shown as Fig. 2c. The $q_{1}$ and $q_{2}$ of the point that is the closest to the origin of coordinates are selected as the best controller parameters of this condition. The best controller parameters under various working conditions are shown in Fig. 2a,b.

According to Fig. 2a,b, with the increasing of vehicle speed *V* or road roughness grade index *d*, or the decreasing of sprung mass $m_{H}$, $q_{1}$ is decreased and $q_{2}$ is increased. This is because with *V* and *d* increasing, and $m_{H}$ decreasing, the sprung mass acceleration $\left| {\ddot{x}_{H} } \right|$ has an increasing tendency which deteriorates the riding comfort. Based on this, decreasing $q_{1}$ and increasing $q_{2}$ can improve the suspension deflection (i.e.$\left| {x_{Hf} - x_{f} } \right|$ and $\left| {x_{Hr} - x_{r} } \right|$)and reduce the sprung mass acceleration (i.e.$\left| {\ddot{x}_{H} } \right|$), which can improve the riding comfort.

### Adaptive suspension controller modelling

In this part, according to the optimal results of $q_{1}$ and $q_{2}$ shown in Fig. 2, the fuzzy adaptive suspension controller is proposed and the parameters of the IMMAKF sub-models are calculated.

Based on the optimization results of Fig. 2, the fuzzy adaptive suspension controller model is established. Compared with the vehicle speed *V* and road grade *d*, the sprung mass $m_{H}$ is difficult to be directly measured. Since sprung mass acceleration $\ddot{x}_{H}$ is influenced by the sprung mass $m_{H}$, the fuzzy controller is built with vehicle speed *V*, road roughness grade index *d* and sprung mass acceleration $\left| {\ddot{x}_{H} } \right|$ as inputs, $q_{1}$ and $q_{2}$ as output respectively.

**Figure 3:**
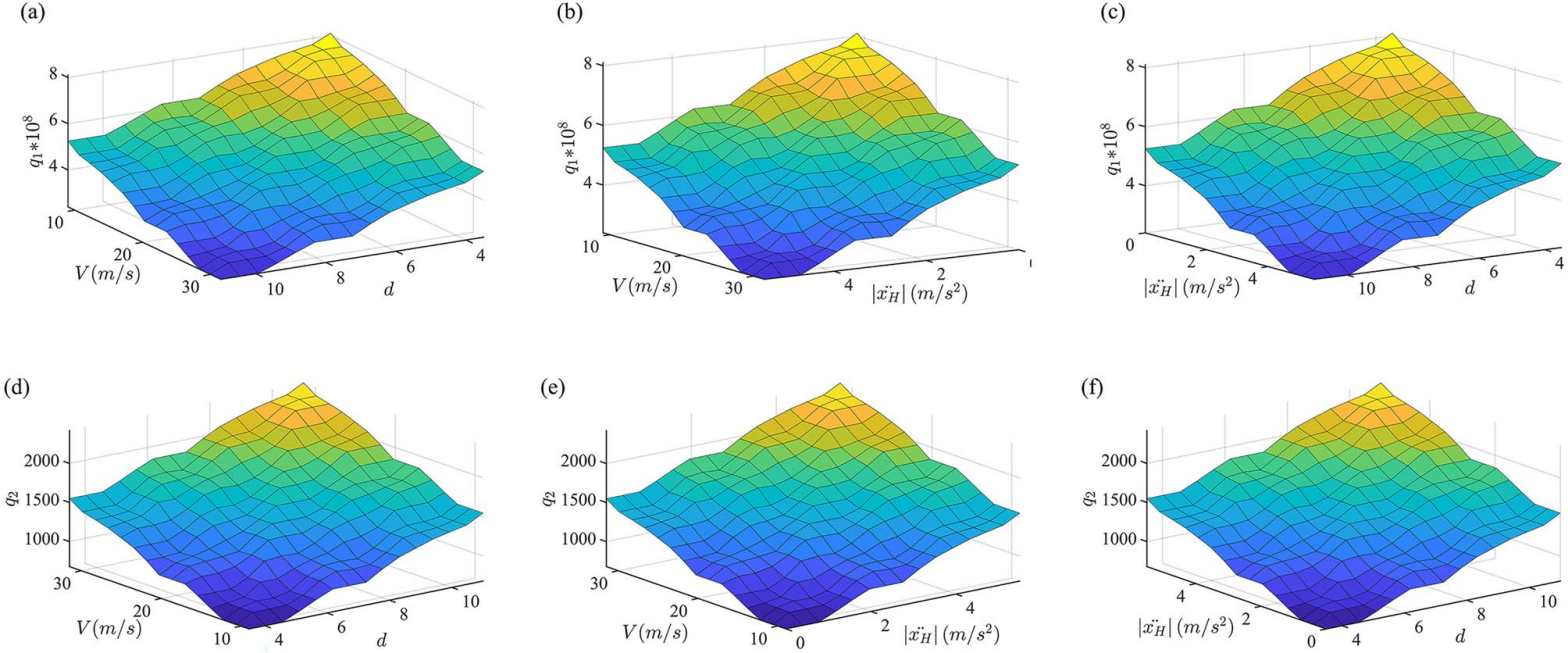
The fuzzy control relationships. (**a**, **b**, **c**) the relationship between *d*, *V*,$\left| {\ddot{x}_{H} } \right|$ and $q_{1}$; (**d**, **e**, **f**) the relationship between *d*, *V*,$\left| {\ddot{x}_{H} } \right|$ and $q_{2}$.

The fuzzy subset of input variable is divided into: VS (very small), S (small), LS (little small), M (medium), LB (little big), B (big), VB (very big), and the fuzzy subset of output variable is divided into: S1, S2, S3, S4, S5, M, B1, B2, B3, B4, B5. Centroid style is set as fuzzy control mode. The fuzzy control relationships are shown in Fig. 3.

According to Fig. 3, with vehicle speed *V*, road grade index *d* and absolute value of vehicle acceleration $\left| {\ddot{x}_{H} } \right|$ increase, $q_{1}$ is decreased and $q_{2}$ increased, which makes the body acceleration $\ddot{x}_{H}$ always be maintained at a small trend to improve the vehicle ride comfort.

**Figure 4:**
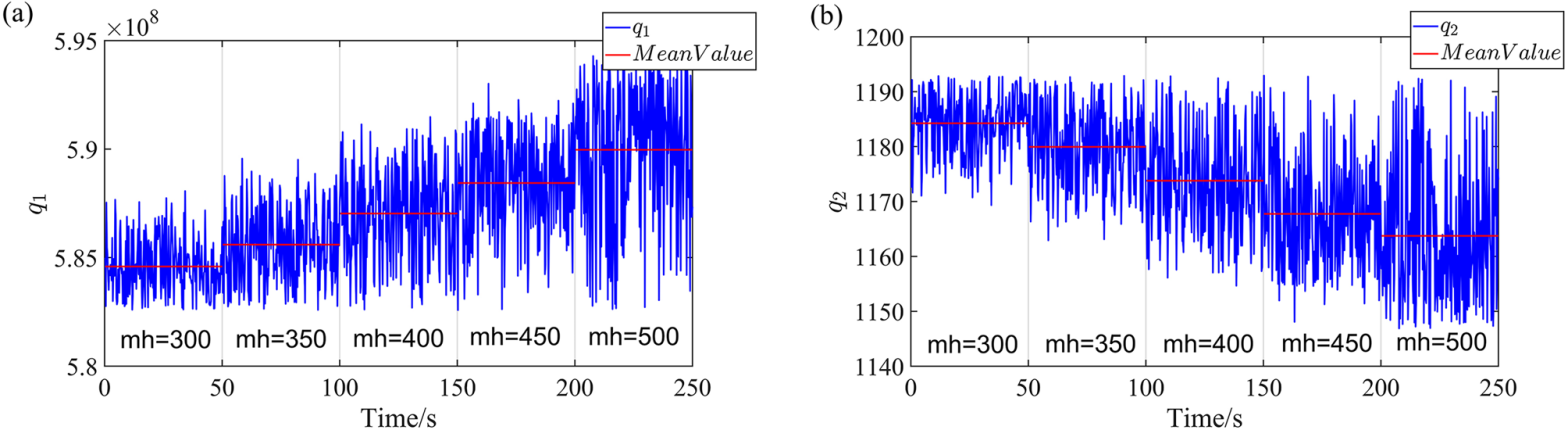
Simulation results of q1 and q2.

The established fuzzy adaptive suspension model is simulated to calculate the parameters of IMMAKF sub-models. As an example, the simulation condition is ISO C road, the vehicle speed is 20 ${\text{m s}}^{{ - 1}}$, the simulation duration is 250 s, and the sprung mass changes as 300–350-400–450-500 kg every 50 s. $q_{1}$ and $q_{2}$ are extracted in the simulation process, as shown in Fig. 4:

According to Fig. 4, controller parameters are automatically adjusted by the fuzzy adaptive suspension model according to the working conditions. In Fig. 4, the mean value of $q_{1}$ and $q_{2}$(as shown in the red lines in Fig. 4) are changed with sprung mass $m_{H}$.When the sprung mass $m_{H}$ increases, the mean value of $q_{1}$ increases but $q_{2}$ decreases. Therefore, the sprung mass $m_{H}$ is an important factor to affect controller parameters. The mean values of $q_{1}$ and $q_{2}$ under various working conditions are calculated as parameters of sub-models of the IMMAKF state observer, as shown in Table A.1.

## Suspension IMMAKF state estimation theory

In this section, the suspension IMMAKF state estimation and control theory is provided in details.

In practice, the suspension control effect is influenced by external disturbances (like changing road roughness) and parameter uncertainties (like changing vehicle speed and sprung mass). Within these conditions, the vehicle speed can be directly measured by the on-board sensor, however the road roughness grade, sprung mass and suspension state parameters like suspension deflection and sprung mass vertical velocity are hard to be measured directly.

The vibration effect of suspension is affected by the estimation precision of each state parameter. At present, Karman Filter (KF) is mostly used as state observer to calculate the real-time state. The ordinary Kalman filter has a high requirement on the accuracy of the system modelling. Since the state of adaptive suspension is changed with the change of working conditions, the ordinary Kalman filter observer is hard to satisfy the high accuracy order of state estimation of adaptive suspensions.

Since the optimal state of the vehicle under various working conditions can be preset (as shown in Table A.1), the suspension state observer of interactive multiple model adaptive Kalman filter (IMMAKF) is proposed by combining adaptive Kalman filter (AKF) and interacting multiple model Kalman filter (IMMKF) to improve the estimation accuracy of adaptive suspension.

### State estimation theory of IMMAKF

In this part, the IMMAKF suspension state observer is proposed.

**Figure 5:**
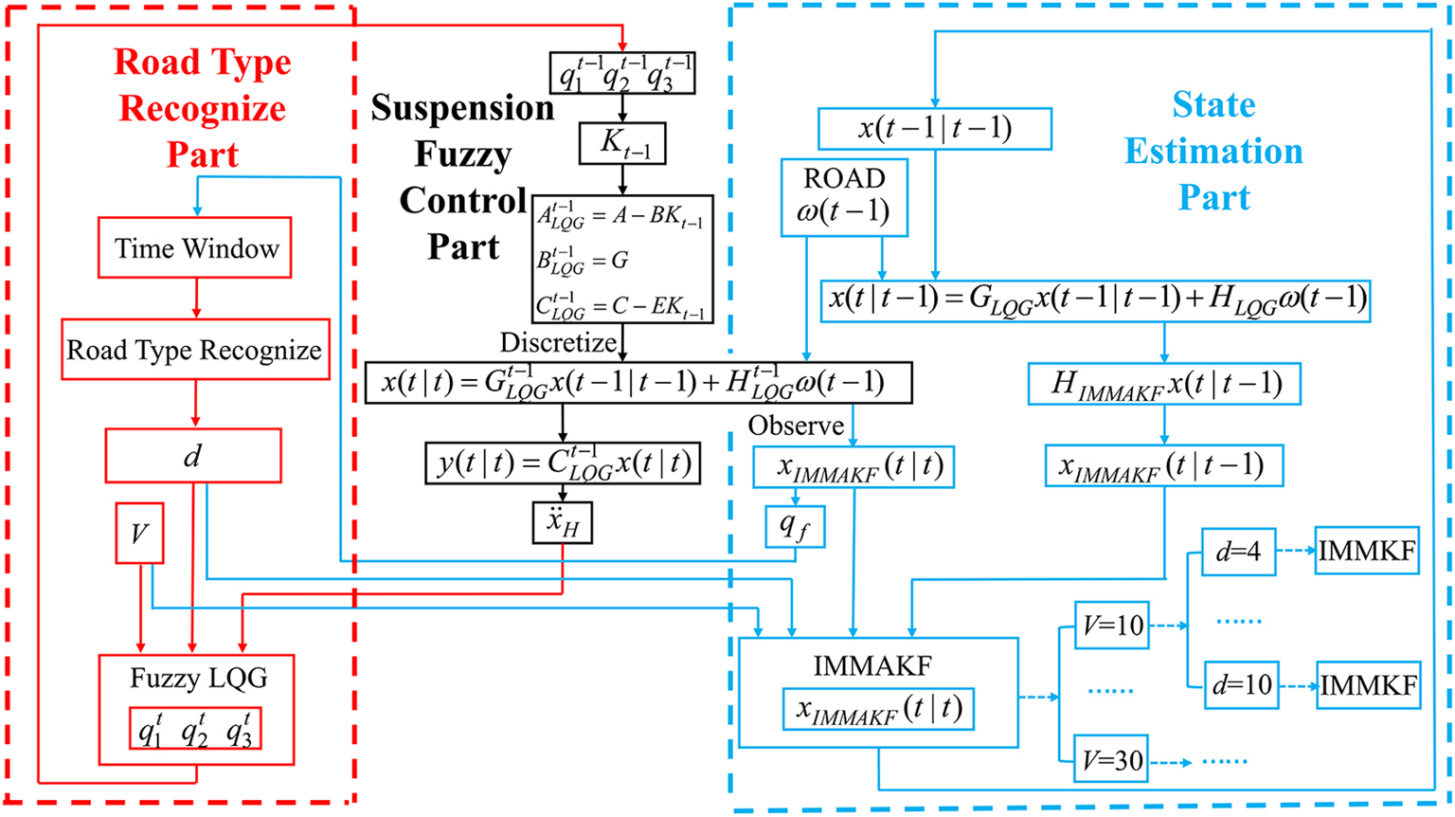
Flow chart of observation and control of IMMAKF.

The IMMAKF observer is proposed based on Table A.1. Firstly, the vehicle speed *V* is determined by the onboard sensor, and the road grade index *d* is calculated according to the state observation results. Then the adaptive suspension is controlled by the fuzzy control model and the suspension state is estimated by IMMAKF observer. The control and observation process are shown in Fig. 5.

The steps of IMMAKF state estimation are shown as Step 1 to 5.

*Step 1*: Model interaction.

The IMMAKF state observer is formed with *s* IMMKF models. Each IMMKF model has *r* sub-models. All sub-models are Markov processes. Each IMMKF model corresponds to a grade of road, and each IMMKF sub-model corresponds to a sprung mass.

Set $x_{k - 1}^{d,i}$ as the original system state of sub-model *i* of the IMMKF model with road roughness grade index *d* at the step *t* = *k-*1, $P_{k - 1}^{d,i}$ is the covariance matrix, and $\mu_{k - 1}^{d,i}$ is the probability of model *i*.

After model interaction, the initial conditions of IMMAKF are $x_{k - 1}^{d,i,0}$ and $P_{k - 1}^{d,i,0}$, and can be calculated as shown in Eq. (18) and (19), respectively.

18 $$ x_{k - 1}^{d,i,0} = \sum\limits_{j = 1}^{r} {x_{k - 1}^{d,j} \mu_{k - 1}^{d,ji} } $$

19 $$ P_{k - 1}^{d,i,0} = \sum\limits_{j = 1}^{r} {\mu_{k - 1}^{d,ji} \left[ {P_{k - 1}^{d,j} + (x_{k - 1}^{d,j} - x_{k - 1}^{d,i,0} )\left( {x_{k - 1}^{d,j} - x_{k - 1}^{d,i,0} } \right)^{T} } \right]} $$

where

20 $$ \mu_{k - 1}^{d,ji} = \frac{{p_{ji}^{d} \mu_{k - 1}^{d,j} }}{{\sum {p_{ji}^{d} \mu_{k - 1}^{d,j} } }} $$

In Eq. (20),$\mu_{k - 1}^{d,ji}$ is the mix probability of model *j* transferred to model *i* of the IMMKF model with road grade index *d* at the step *t* = *k*-1, and $p_{ji}^{d}$ is the transition probability matrix from model *j* to model* i*.

*Step 2*: Kalman filter.

$x_{k - 1}^{d,i,0}$ and $P_{k - 1}^{d,i,0}$ calculated in the Step 1 are used for state prediction and prior covariance estimation.

State prediction equation is shown as Eq. (21).

21 $$ x_{k|k - 1}^{d,i} = A_{i}^{d} x_{k - 1}^{d,i,0} + B_{i}^{d} u_{k - 1}^{d} $$

Prior covariance equation is shown as Eq. (22).

22 $$ P_{k|k - 1}^{d,i} = A_{i}^{d} P_{k - 1}^{d,i,0} (A_{i}^{d} )^{T} + Q_{i}^{d} $$

where,$A_{i}^{d}$ and $B_{i}^{d}$ are the state equation of model *i*, and $Q_{i}^{d}$ is the process noise variance matrix.

Then the Kalman gain equation $K_{k}^{d,i}$ is calculated as:

23 $$ K_{k}^{d,i} = \frac{{P_{k|k - 1}^{d,i} H^{T} }}{{HP_{k|k - 1}^{d,i} H^{T} + R}} $$

where, H is the system observation matrix, and R is the measurement noise covariance matrix.

The Kalman filter state is calculated as:

24 $$ x_{k}^{d,i} = x_{k|k - 1}^{d,i} + K_{k}^{d,i} (Z_{k}^{d} - Hx_{k|k - 1}^{d,i} ) $$

where,$Z_{k}^{d}$ is the estimation value at the step *t* = *k*.

The Kalman filter covariance is calculated as:

25 $$ P_{k}^{d,i} = \left( {I - K_{k}^{d,i} H} \right)P_{k|k - 1}^{d,i} $$

*Step 3*: Update model probability.

The observer model is updated by maximum likelihood estimation. By calculating the similarity between the current model and the current target state, the most suitable weight of the current tracking model is given at step *t* = *k*. The most matched maximum likelihood function of model* i* is calculated as follows:

26 $$ \Lambda_{k}^{d,i} = \frac{1}{{\sqrt {2\pi^{N} \det \left| {S_{k}^{d,i} } \right|} }}\exp \left[ { - 0.5(d_{k}^{d,i} )^{T} (S_{k}^{d,i} )^{ - 1} (d_{k}^{d,i} )} \right] $$

where

27 $$ d_{k}^{d,i} = z_{k} - H_{i} x_{k|k - 1}^{d,i} $$

28 $$ d_{k}^{d,i} = z_{k} - H_{i} x_{k|k - 1}^{d,i} $$

The probability of model *i* is updated as:

29 $$ \mu_{k}^{d,i} = \frac{1}{c}\Lambda_{k}^{d,i} \sum {p_{ji}^{d} \mu_{k - 1}^{d,j} } $$

where

30 $$ c = \frac{{\Lambda_{k}^{d,i} }}{{\sum {\Lambda_{k}^{d,i} } }}\sum {p_{ji}^{d} \mu_{k - 1}^{d,j} } $$

*Step 4*: Combine model data.

The results of overall state estimation and overall covariance estimation are calculated by Kalman filter states, Kalman filter covariance and updated probability.

Overall state estimation is calculated as Eq. (31).

31 $$ x_{k}^{d} = \sum\limits_{i = 1}^{r} {x_{k}^{d,i} \mu_{k}^{d,i} } $$

Overall covariance estimation is calculated as Eq. (32).

32 $$ P_{k}^{d} = \sum\limits_{i = 1}^{r} {\mu_{k}^{d,i} \left[ {P_{k}^{d,i} + \left( {x_{k}^{d,i} - x_{k}^{d} } \right)\left( {x_{k}^{d,i} - x_{k}^{d} } \right)^{T} } \right]} $$

The model *d* of Eqs. (18) to (32) is determined by the state estimation of the road profile (i.e.$q_{f}$ and $q_{r}$).

From Step 4, the state parameters are calculated by IMMAKF observer. The road roughness grade is calculated by Step 5.

*Step 5*: Determine the current road roughness grade.

The road grade index *d* is calculated by the power spectral density (PSD) of $q_{f}$ and $q_{r}$ through state observation, as the reference of mode switching for suspension controller and observer. In this paper, road roughness grade is calculated by $q_{f}$. The road displacement unevenness $q(l)$ is calculated by $q_{f}$ and driving length *l*. Since sampling time and vehicle speed *V* are fixed, *q(l)* can be transformed into *q(n)*.

The average power of *q(n)* is:

33 $$ P = \frac{1}{N}\sum\limits_{n = 0}^{N - 1} {\left| {q(n)} \right|^{2} } $$

where *N* is the total number of points in a sampling process.

From the discrete Fourier transform:

34 $$ Q_{m} = \sum\limits_{n}^{N - 1} {q(n)e^{{ - jmn\frac{2\pi }{N}}} } $$

According to Parseval theory^31^:

35 $$ \sum\limits_{n = 0}^{N - 1} {\left| {q(n)} \right|^{2} } = \frac{1}{N}\sum\limits_{m = 0}^{N - 1} {\left| {Q_{m} } \right|^{2} } $$

Then Eq. (33) can be expressed as:

36 $$ P = \frac{1}{{N^{2} }}\sum\limits_{m = 0}^{N - 1} {\left| {Q_{m} } \right|^{2} } $$

Therefore, the power spectral density of *q(n)* is:

37 $$ P_{m} = \frac{1}{N}\left| {Q_{m} } \right|^{2} \frac{N}{{f_{s} }} = \frac{1}{{f_{s} }}\left| {Q_{m} } \right|^{2} $$

where, $f_{s}$ is the sampling frequency.

By comparing the calculated $P_{m}$ with standard road roughness grade^32^, the grade of the road can be calculated.

The above steps can be iterated to complete the suspension state observation based on IMMAKF. The specific details of IMMAKF state observation can be described as: IMMKF sub-models with different sprung mass acceleration under various vehicle speed and road grade are combined as IMMAKF observer. When the working condition is changed, the suspension controller switches the state according to the speed, road grade and sprung mass acceleration, and then the IMMAKF observer selects the sub-model (IMMKF) according to the state of the controller system. For IMMAKF, the state of observer is always consistent with the state of the suspension controller.

### Q and R of IMMAKF state observer

The noise generated in state transition is measured by the process noise variance matrix *Q*. *Q* is calculated as Eq. (38).

38 $$ Q = H_{LQG} Q_{RV} Q_{RV}^{T} B_{LQG}^{T} $$

where, $Q_{RV}$ is the noise of input road speed, which can be calculated by approximate statistics in Eq. (39).

39 $$ Q_{RV} = \frac{1}{n}\sum\limits_{i = 1}^{n} {\dot{q}_{i} } $$

where, $\dot{q}_{i}$ is the input road speed at sampling point* i*.

The relationship between $Q_{RV}$ and road roughness grade index *d* described in three degrees polynomial function is:

40 $$ Q_{RV} = 0.00138d^{3} - 0.01516d^{2} + 0.07753d - 0.08562 $$

The SSE of the fitting function is $7.634 \times 10^{ - 4}$, and the R-square is 0.9992. The fitting accuracy is high.

The noise in the observation process is calculated by the measurement noise covariance matrix *R*. Set measurement vector as:

$$ x_{IMMAKF} = \left[ {\begin{array}{*{20}c} {\dot{x}_{H} } & {\dot{\theta }} & {\dot{x}_{f} } & {\dot{x}_{r} } \\ \end{array} } \right]^{\prime} $$

The observation matrix is:

41 $$ H_{IMMAKF} = \left[ {\begin{array}{*{20}c} 1 & 0 & 0 & 0 & {...} & 0 \\ 0 & 1 & 0 & 0 & {...} & 0 \\ 0 & 0 & 1 & 0 & {...} & 0 \\ 0 & 0 & 0 & 1 & {...} & 0 \\ \end{array} } \right] $$

The size of $H_{IMMAKF}$ is $4 \times 9$.

*R* is dependent on the sensor accuracy and road conditions. According to^33^, set $R = 0.01^{2}$.

## Suspension state estimation and control based on IMMAKF

In this section, two IMMAKF observers are established. A variable working condition is established based on changing vehicle speed, road roughness grade and sprung mass. Compared with other Kalman filter observers, the precision of IMMAKF observer is verified by simulation and experiment.

### Simulation condition

**Figure 6:**
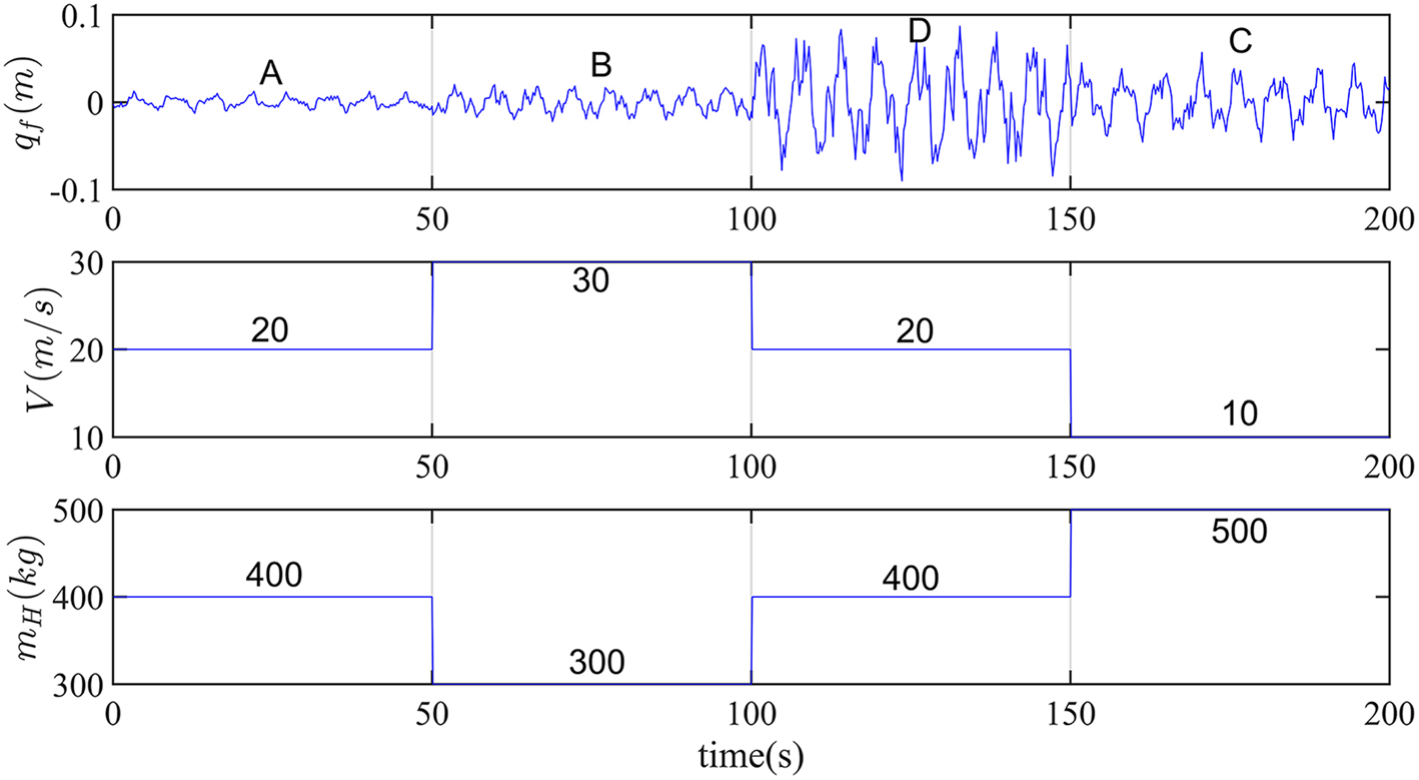
Suspension simulation condition. 0-50 s, V = 20 ${\text{m s}}^{{ - 1}}$,$m_{H} = 400{\text{kg}}$, ISO A road; 50-100 s, V = 30 ${\text{m s}}^{{ - 1}}$,$m_{H} = 300{\text{kg}}$, ISO B road; 100-150 s, V = 20 ${\text{m s}}^{{ - 1}}$,$m_{H} = 400{\text{kg}}$, ISO D road;150-200 s, V = 10 ${\text{m s}}^{{ - 1}}$,$m_{H} = 500{\text{kg}}$, ISO C road.

In practice, the actual working conditions such as vehicle speed, road roughness grade and sprung mass can be changed simultaneously. Based on variable working conditions, referring to Table A.1, the simulation model is established as shown in Fig. 6:

### IMMAKF observer models

Two kinds of IMMAKF observers are proposed by Table A.1.

Five-model IMMAKF observer (IMMAKF5): All the data in Table A.1 are selected as the sub-model of IMMAKF observer. Sub-models of IMMAKF5 cover all working conditions and the observation accuracy is high.

Three-model IMMAKF observer (IMMAKF3): the control parameters with sprung masses of 300, 400 and 500 (kg) in Table A.1 are extracted respectively as sub-models of IMMAKF3 observer. Sub-models of IMMAKF3 cover three main working conditions to maintain observation accuracy.

For comparison, data are selected from Table A.1 as other observers: ordinary Kalman filter (KF), AKF and IMMKF. Among them, the results of 20 ${\text{m s}}^{{ - 1}}$ vehicle speed, ISO C road and 400 kg sprung mass in Table A.1 are selected as the KF. The results of 20 ${\text{m s}}^{{ - 1}}$ vehicle speed, 400 kg sprung mass and all grades of roads are selected as AKF (i.e. for AKF, the vehicle speed and sprung mass are fixed, but road grade can be changed). The results of 20 ${\text{m s}}^{{ - 1}}$ vehicle speed, ISO C road and all of the sprung mass are selected as IMMKF (i.e. for IMMKF, the vehicle speed and the road grade are fixed, but the sprung mass is used as the interacting multiple model for state estimation).

### Road roughness grade recognition time window

Based on Eq. (33) to Eq. (37), the accuracy of road grade identification is influenced by the number of sampling points. The spatial frequency of road roughness is between $\left[ {0.011,2.83} \right]{\text{m}}^{{ - 1}}$. The minimum identification frequency *dn* is calculated as Eq. (42).

42 $$ dn = \frac{1}{l} \le 0.011 $$

Then,$$l \ge 91{\text{m}}$$.

The minimum identification sampling step number is set as $n_{\min }$, then

43 $$ n_{\min } \;\Delta t\;V_{\min } \ge 91{\text{m}} $$

where,$\Delta t$ is sampling interval time of adjacent sampling points, and *V* is vehicle speed. In this paper, $\Delta t = 0.01s$ and $V_{\min } = 10{\text{m/s}}$,then $n_{\min } \ge 910$.

The road grade roughness shown in Fig. 6 is estimated by IMMAKF5. Set *n* as the step number of road roughness grade recognition window. Different road grade estimation accuracy of different *n* is shown as Table 2.

**Table 2:** Road grade estimation accuracy.

Sampling step n	ISO A/%	ISO B/%	ISO D/%	ISO C/%
910	85.00	85.00	80.00	85.00
1000	85.00	85.00	87.50	90.00
1100	90.00	90.00	90.00	100.00
1200	100.00	100.00	95.00	100.00
1250	100.00	100.00	100.00	100.00

In Table 2, the higher the number of sampling steps *n*, the higher the accuracy of road grade recognition, but the longer the distance for the vehicle to derive. Based on the accuracy and the driving time, the step number of road roughness grade recognition window is selected as 1250.

### Comparison of simulation results

**Figure 7:**
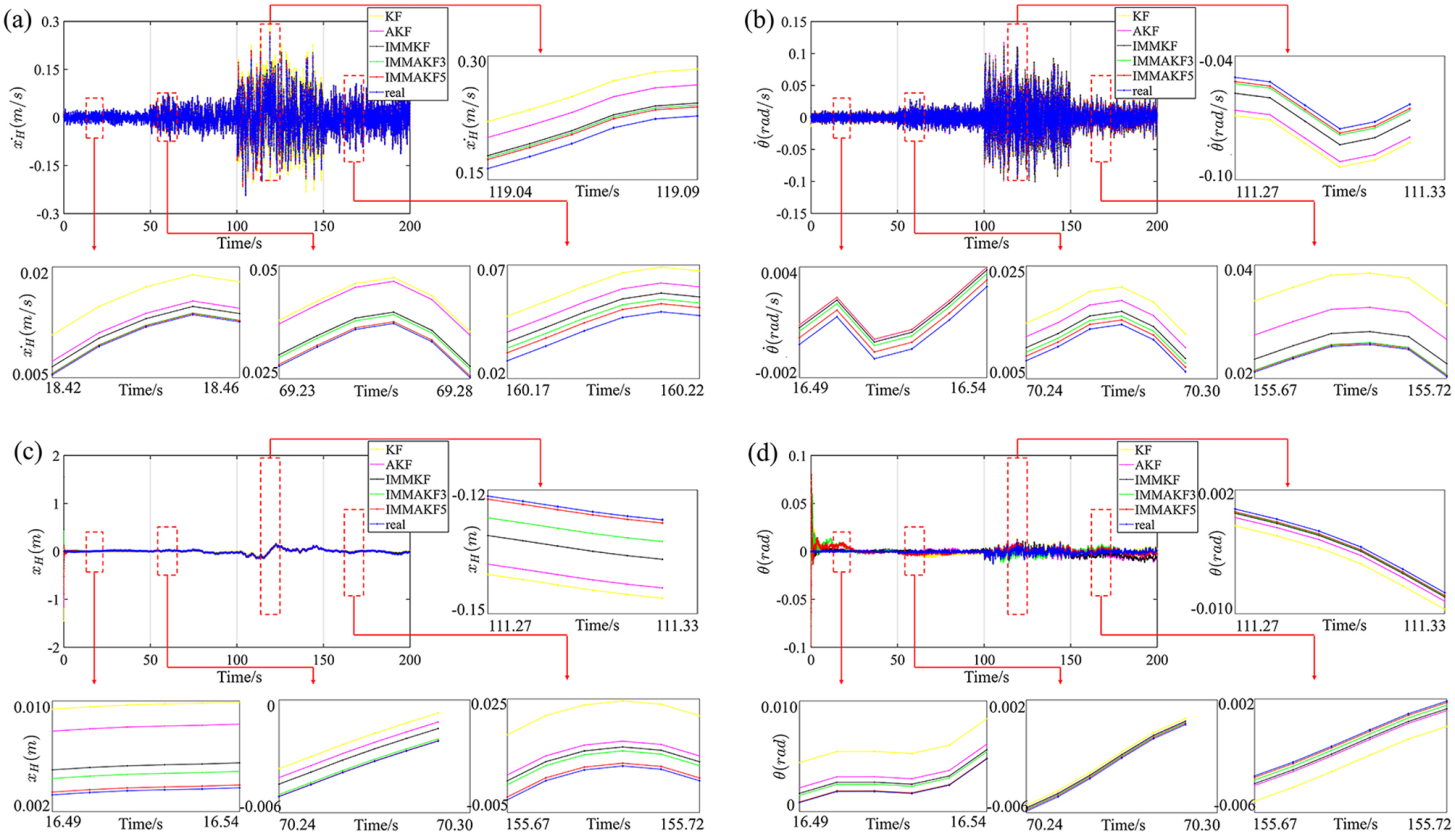
Model simulation and comparison results: (**a**) results of $\dot{x}_{H}$ (**b**) results of $\dot{\theta }$ (**c**) results of $x_{H}$ (**d**) results of $\theta$.

The IMMAKF5, IMMAKF3, KF, AKF and IMMKF observers are applied to the state estimation of adaptive suspension. The initial road roughness grade of IMMAKF and AKF is determined as A grade (i.e. the initial* d* = 4). The comparison results are shown in Fig. 7:

According to Fig. 7, for all working conditions, the estimation results of IMMAKF5 is the closest to real results, followed by IMMAKF3. Since state equations of adaptive suspensions are changed with the change of vehicle speed, road roughness grade and sprung mass, the state estimation accuracy of fixed state observer is low. Therefore, in Fig. 7, the estimation accuracy of KF is the worst. Compared with KF, both AKF and IMMKF can partly follow the current suspension state (i.e. road grade and sprung mass), which makes the estimation accuracy of AKF and IMMKF lower than IMMAKF but higher than KF.

The simulation error of each observer is calculated as shown in Eq. (44).

44 $$ E_{s} = \frac{1}{n}\sum\limits_{1}^{n} {\frac{{\left| {x_{ireal} - x_{iobs} } \right|}}{{\left| {x_{ireal} } \right|}}} \times 100\% $$

where, $E_{s}$ is the simulation error, $x_{ireal}$ is the real value of $x_{i}$, $x_{iobs}$ is the observation value of $x_{i}$, *n* is the total number of the sampling points in the simulation, and *i* is the number of coefficients of state vector *x*.

The simulation errors of each coefficient of state vector *x* are shown in Table 3.

**Table 3:** Comparison of simulation error.

Working Condition	Model	Simulation error/%									
		$\dot{x}_{H}$	$\dot{\theta }$	$\dot{x}_{f}$	$\dot{x}_{r}$	$x_{H}$	$\theta$	$x_{f}$	$x_{r}$	$q_{f}$	$q_{r}$
Total	IMMAKF5	4.73	4.90	1.86	1.79	12.78	2.05	14.75	10.54	4.88	5.95
	IMMAKF3	5.06	4.91	1.90	1.83	15.46	2.21	15.01	10.82	5.05	6.17
	IMMKF	10.55	9.55	8.15	7.71	15.91	2.54	15.82	13.06	15.78	13.10
	AKF	10.96	9.58	8.24	7.80	17.28	3.26	18.55	16.30	19.23	16.44
	KF	11.88	8.96	9.01	8.70	20.21	3.31	20.39	19.20	20.31	19.52
Grade A	IMMAKF5	5.10	6.24	3.6	3.51	16.70	4.04	13.27	15.98	4.95	7.04
	IMMAKF3	6.08	6.23	3.91	3.63	17.54	4.06	14.39	20.54	5.87	7.92
	IMMKF	15.55	10.27	10.79	10.49	17.44	5.31	15.17	28.79	17.20	10.89
	AKF	15.97	10.06	10.80	10.45	18.72	5.67	19.52	28.75	17.84	16.78
	KF	17.26	10.04	10.84	10.55	25.09	9.12	20.58	29.57	24.74	20.25
Grade B	IMMAKF5	3.13	3.56	1.55	1.70	17.56	1.24	21.71	12.54	7.16	7.02
	IMMAKF3	3.16	3.91	1.66	1.89	21.60	1.72	22.65	14.44	7.53	8.19
	IMMKF	9.46	8.37	10.07	8.70	22.02	3.86	26.34	16.00	16.48	15.89
	AKF	10.66	8.74	10.48	8.73	23.08	3.41	27.74	19.61	16.97	19.90
	KF	12.50	9.63	11.36	9.64	23.43	4.15	28.69	20.01	21.42	20.45
Grade C	IMMAKF5	5.71	6.12	1.30	1.34	11.83	2.37	10.54	9.84	4.10	5.62
	IMMAKF3	5.90	6.35	1.31	1.35	15.01	2.43	11.84	11.61	4.14	6.56
	IMMKF	9.71	8.19	7.40	7.17	17.75	2.71	18.90	16.64	15.70	16.30
	AKF	9.16	8.15	7.36	7.02	17.98	2.75	18.96	18.39	18.68	18.36
	KF	10.17	8.97	7.91	7.92	18.88	4.51	19.49	18.82	26.53	19.20
Grade D	IMMAKF5	4.92	3.54	0.70	0.47	7.21	3.72	6.72	5.14	2.29	2.55
	IMMAKF3	5.14	3.55	0.71	0.50	8.74	4.46	10.58	5.16	3.52	2.81
	IMMKF	7.53	9.95	4.45	4.26	10.75	5.68	13.12	7.67	13.24	7.70
	AKF	6.27	9.58	4.45	4.11	12.29	7.14	19.46	9.66	20.16	20.91
	KF	10.89	7.94	6.17	5.88	14.41	8.20	19.81	17.74	23.77	16.87

Where, IMMAKF5 is results of five-model IMMAKF observer, IMMAKF3 is results of three-model IMMAKF observer. Total is the whole simulation condition. Grade A is the simulation condition of grade A road in Fig. 6.

In Table 3, compared with other observers, the estimation error of IMMAKF3 and IMMAKF5 observers are the smallest. Within the two IMMAKF observers, IMMAKF5 has the highest accuracy. Among the four measurement vector coefficients ($$\left[ {\begin{array}{*{20}c} {\dot{x}_{H} } & {\dot{\theta }} & {\dot{x}_{f} } & {\dot{x}_{r} } \\ \end{array} } \right]^{\prime}$$), for the whole simulation process, compared with IMMKF, AKF and KF, the estimation error of $\dot{x}_{H}$ in IMMAKF5 is reduced by 55.17%, 56.84% and 60.19% respectively; $\dot{\theta }$ is reduced by 48.69%, 48.85% and 45.31% respectively; $\dot{x}_{f}$ is reduced by 77.18%, 77.43% and 79.36% respectively;$\dot{x}_{r}$ is reduced by 76.78%, 77.05% and 79.43% respectively. The trend of simulation results on each grade of road is similar to the total simulation process. For other state coefficients, the accuracy of the two IMMAKF observers is also better than other observers, which verifies the superiority of the IMMAKF observer in complex working conditions.

**Figure 8:**
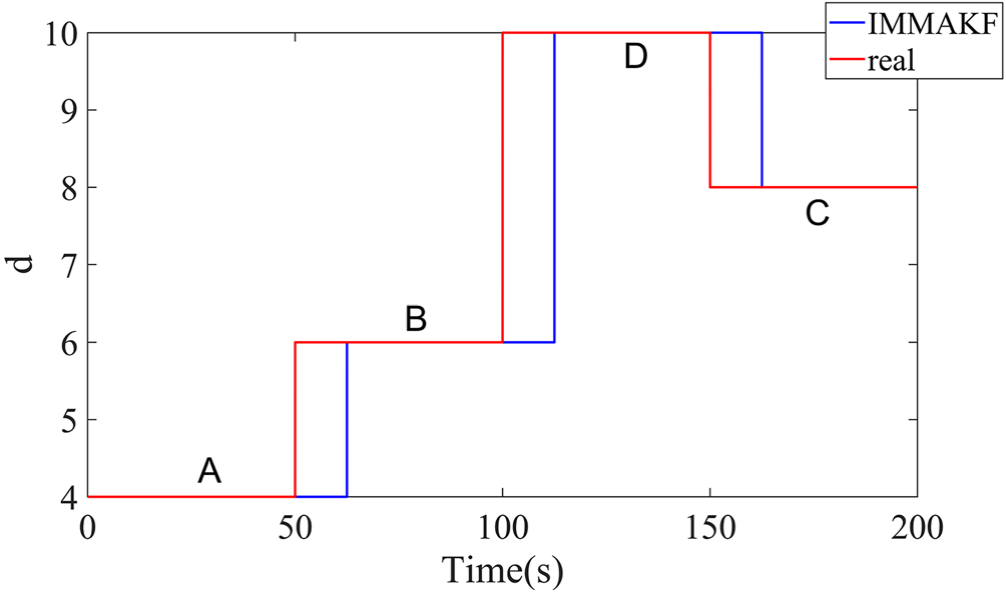
Road grade recognition results of IMMAKF.

During the simulation, the road grade is calculated by IMMAKF observer. The road grade recognition results for IMMAKF5 and IMMAKF3 are shown in Fig. 8:

In Fig. 8, the road roughness grade recognition results always lag behind the real road in time domain, and the lag time is just the length of a time window. Since the road grade recognition depends on sampling of $q(l)$, the system can’t immediately calculate the road grade.

**Figure 9:**
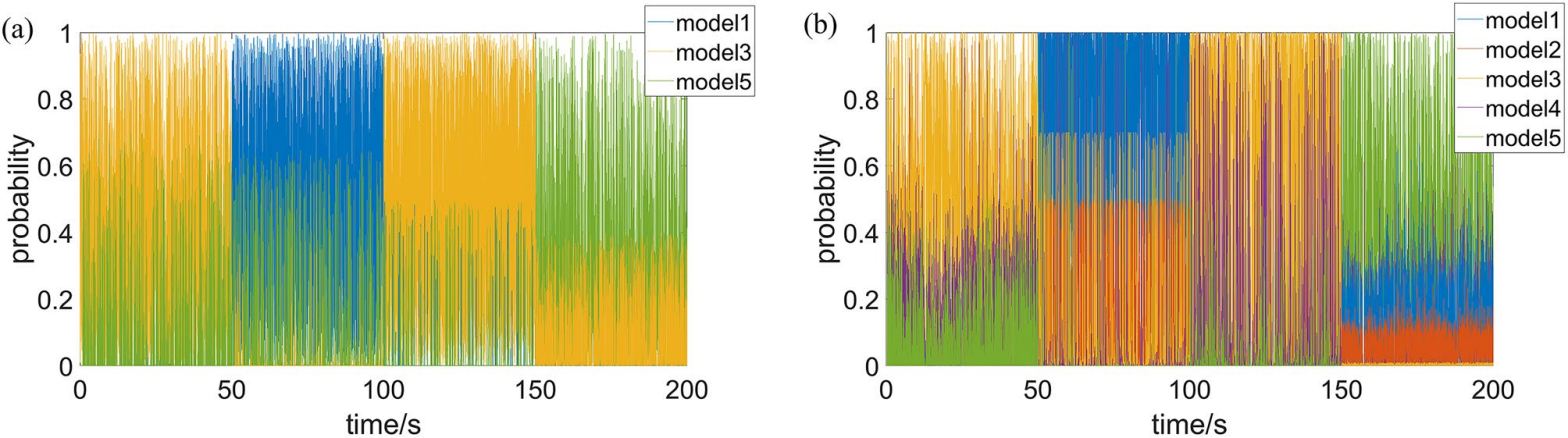
Model probability of IMMAKF. (**a**) Model probability of IMMAKF3 (**b**) Model probability of IMMAKF5. For model 1 to model 5, the sprung mass $m_{H}$ is 300, 350, 400, 450, 500 kg, respectively.

During the simulation, the model interaction probability of IMMAKF3 and IMMAKF5 are shown as Fig. 9:

According to Fig. 9, during the model interaction process, IMMAKF always has a sub-model with the maximum probability, which is related to the current model state, i.e. the sub-model with the maximum interaction probability in IMMAKF algorithm is the current system state model, which can be used to estimate the current sprung mass. For Fig. 9, the *n* steps time window is used to estimate the sprung mass of the suspension model, and the accuracy of sprung mass estimation is shown in Table 4:

**Table 4:** Sprung mass estimation accuracy of IMMAKF observers.

Sampling step *n*	Model	Condition1	Condition2	Condition3	Condition4
1	IMMAKF3	68.10%	84.40%	89.60%	46.20%
	IMMAKF5	48.00%	66.80%	57.40%	40.10%
50	IMMAKF3	92.90%	100.00%	100.00%	55.50%
	IMMAKF5	69.80%	98.00%	91.80%	65.60%
100	IMMAKF3	97.90%	100.00%	100.00%	85.30%
	IMMAKF5	73.50%	100.00%	97.80%	77.50%
500	IMMAKF3	100.00%	100.00%	100.00%	100.00%
	IMMAKF5	100.00%	100.00%	100.00%	100.00%

In Table 4, the larger the sampling step of the time window, the higher the model recognition accuracy, but the longer the model sampling time, and the longer the vehicle driving distance. When the sampling step is 500, the model has the highest accuracy and sampling time is also suitable, so 500 steps is considered as the optimal step size for sprung mass estimation.

According to Fig. 9 and Table 4, IMMAKF observers can not only ensure high estimation accuracy under variable working conditions, but also determine the sprung mass through analyzing model interaction probability.

### Experiment verification

**Figure 10:**
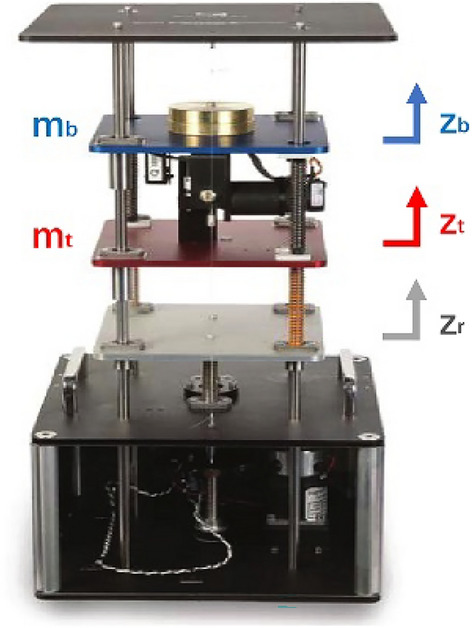
Suspension experiment platform.

The correctness of IMMAKF state observer is verified by the suspension experiment platform. The platform is shown in Fig. 10.

**Figure 11:**
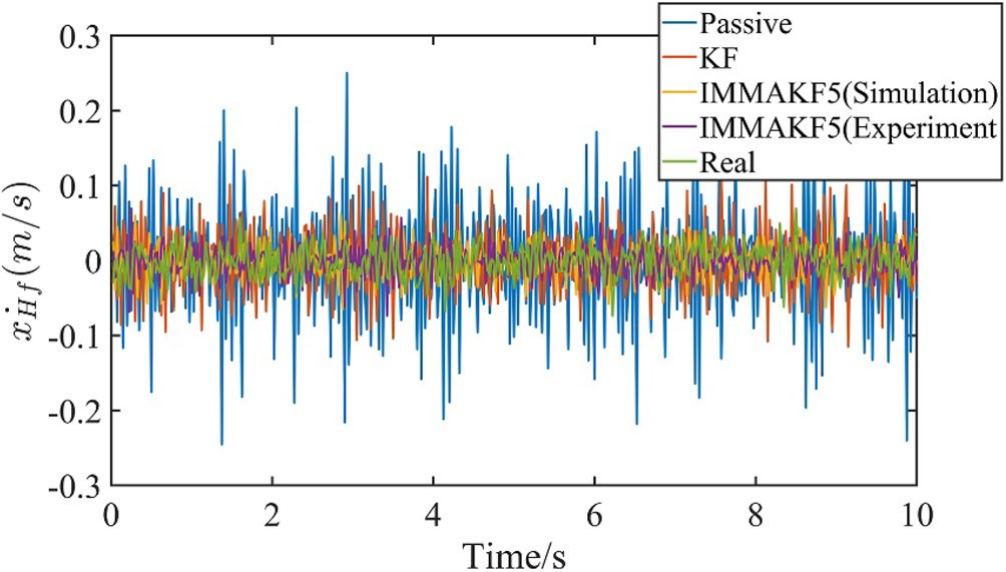
Suspension experiment results of $\dot{x}_{Hf}$.

Since the platform is the quarter vehicle model, the suspension is used to simulate the front wheel. For the platform, the sprung mass is 2.45 kg, unsprung mass is 1 kg, the suspension stiffness is 900 N/m and the tire stiffness is 2500N/m. For the experiment, the road excitation is ISO A grade. The total experiment time is 10 s and the time interval is 0.02 s. The vehicle speed is 20 m/s. The experiment results of ordinary KF (i.e. 20 m/s vehicle speed, ISO C road and 2.45 kg sprung mass) and simulation results of IMMAKF5 are selected for comparison. To show the vibration effect (i.e. ride comfort and road handling stability), the passive suspension with damping of 7.5 Ns/m is used for verification (the passive suspension doesn’t have observers, the results of passive suspension are obtained by sensors and calculated as comparison). The experiment results of $\dot{x}_{Hf}$ are shown in Fig. 11.

The simulation results comparison is shown in Table 5.

**Table 5:** Simulation results comparison.

Parameter type	Parameters	Model			
		IMMAKF5 (Simulation)	IMMAKF5 (Experiment)	KF	Passive
Observation error/%	$\dot{x}_{Hf}$	4.61	5.27	10.42	–
	$\dot{x}_{f}$	1.96	2.23	8.81	–
Vibration values	$\ddot{x}_{Hf}$	0.2862	0.2735	0.3204	0.7339
	$x_{Hf} - x_{f}$	0.0033	0.0031	0.0035	0.0121

In Fig. 11, the experiment results of IMMAKF5 is close to real values and simulation values, which verifies the accuracy of the IMMAKF5. In Table 5, the experiment errors of IMMAKF5 is smaller than KF, which verifies the superiority of IMMAKF.

In Table 5, compared with the results of KF and passive suspension, the sprung mass acceleration of IMMAKF5 is improved by 14.64% and 62.73% respectively; the suspension deflection is improved by 11.43% and 74.38% respectively, which verifies that the IMMAKF observer can improve the ride comfort and balance the handling stability of the vehicle.

## Conclusions

In this paper, the IMMAKF suspension state observer is proposed for the state observation of the adaptive suspension model under variable working conditions (vehicle speed, road roughness grade and sprung mass). Firstly, the adaptive suspension controller is established. The LQR controller parameters under variable working conditions are optimized by NSGA-II algorithm. Referring to the optimal results, the fuzzy adaptive suspension controller is presented. The adaptive suspension can automatically change the optimal controller parameters by working conditions. Furthermore, the IMMAKF suspension state estimation theory is discussed in detail. Based on IMMKF and AKF, an IMMAKF suspension state observer is established, and the theoretical equations of the algorithm are derived. Lastly, the simulation and experiment results show that, compared with state observers of KF, AKF and IMMKF, the accuracy of the IMMAKF state observer is the highest and the ride comfort of IMMAKF is improved. Except for high estimation accuracy, the road roughness grade and sprung mass can be calculated by IMMAKF observer. Therefore, IMMAKF is effective for high suspension estimation accuracy and the adaptive control on complex conditions.

Note that the proposed method is a suspension state observer, which is established based on the typical working conditions, so the application of this proposed method is limited. The state estimation schema of full car suspension model under more complex working conditions such as the longitudinal vehicle speed with acceleration and the vehicle driving on slope roads, will be investigated in our future study. In addition, the road roughness grade identification by visual sensors will also be considered in the future study.

### Supplementary Information

Supplementary Table 1.

## Data Availability

All data generated or analysed during this study are included in this published article.
