# Supplementary material for: Adaptive suspension state estimation based on IMMAKF on variable vehicle speed, road roughness grade and sprung mass condition
Source: Sci Rep. 2024 Jan 19;14:1740. doi: 10.1038/s41598-023-49766-y (PMC10799087; doi:10.1038/s41598-023-49766-y)
Supplement: Supplementary file 1 — Supplementary Table 1. [file 41598_2023_49766_MOESM1_ESM.docx]

**Appendix A**

**Table A.1.** Parameters of sub-models of IMMAKF

| V/$\text{m s}^{\text{-1}}$ | **Road Grade** | **Parameter** | **$m_{H}\text{/kg}$** | | | | |
| --- | --- | --- | --- | --- | --- | --- | --- |
|  |  |  | **300** | **350** | **400** | **450** | **500** |
| 10 | A | $q_{1}\times10^{8}$ | 8.7505 | 8.7506 | 8.7507 | 8.7513 | 8.7519 |
|  |  | $q_{2}$ | 368.0566 | 367.7465 | 367.4101 | 367.0190 | 366.6217 |
|  | B | $q_{1}\times10^{8}$ | 7.8585 | 7.8670 | 7.8679 | 7.8690 | 7.8697 |
|  |  | $q_{2}$ | 506.0015 | 505.9422 | 505.7045 | 505.6369 | 505.6282 |
|  | C | $q_{1}\times10^{8}$ | 7.1760 | 7.1788 | 7.1812 | 7.1837 | 7.1879 |
|  |  | $q_{2}$ | 848.3294 | 847.6461 | 847.3714 | 846.7037 | 845.3779 |
|  | D | $q_{1}\times10^{8}$ | 6.0764 | 6.0894 | 6.0975 | 6.1039 | 6.1114 |
|  |  | $q_{2}$ | 1191.1441 | 1187.9407 | 1185.7641 | 1184.1012 | 1182.6103 |
| 15 | A | $q_{1}\times10^{8}$ | 8.2402 | 8.2493 | 8.2594 | 8.2706 | 8.2822 |
|  |  | $q_{2}$ | 513.1373 | 512.3291 | 511.5950 | 510.9210 | 510.3448 |
|  | B | $q_{1}\times10^{8}$ | 7.5217 | 7.5286 | 7.5344 | 7.5393 | 7.5426 |
|  |  | $q_{2}$ | 752.9736 | 750.5810 | 747.9987 | 745.0537 | 741.8937 |
|  | C | $q_{1}\times10^{8}$ | 6.5663 | 6.5828 | 6.5993 | 6.6156 | 6.6305 |
|  |  | $q_{2}$ | 998.3249 | 993.3200 | 988.6422 | 984.1442 | 980.0171 |
|  | D | $q_{1}\times10^{8}$ | 5.4067 | 5.4368 | 5.4632 | 5.4889 | 5.5146 |
|  |  | $q_{2}$ | 1359.0144 | 1350.3183 | 1341.5175 | 1334.2392 | 1327.2153 |
| 20 | A | $q_{1}\times10^{8}$ | 7.7341 | 7.7375 | 7.7399 | 7.7416 | 7.7428 |
|  |  | $q_{2}$ | 721.6072 | 718.1422 | 714.6989 | 711.4886 | 708.6362 |
|  | B | $q_{1}\times10^{8}$ | 6.8294 | 6.8439 | 6.8576 | 6.8701 | 6.8810 |
|  |  | $q_{2}$ | 947.4781 | 946.1278 | 945.1535 | 944.5586 | 944.1664 |
|  | C | $q_{1}\times10^{8}$ | 5.8444 | 5.8598 | 5.8749 | 5.8873 | 5.8975 |
|  |  | $q_{2}$ | 1186.7618 | 1179.5114 | 1173.3550 | 1168.0820 | 1163.6901 |
|  | D | $q_{1}\times10^{8}$ | 4.7067 | 4.7321 | 4.7561 | 4.7783 | 4.7970 |
|  |  | $q_{2}$ | 1539.2200 | 1529.6539 | 1521.0440 | 1513.2776 | 1506.0805 |
| 25 | A | $q_{1}\times10^{8}$ | 6.7855 | 6.7887 | 6.7911 | 6.7927 | 6.7938 |
|  |  | $q_{2}$ | 860.8446 | 858.5406 | 856.6538 | 855.1604 | 854.0388 |
|  | B | $q_{1}\times10^{8}$ | 5.9549 | 5.9603 | 5.9641 | 5.9667 | 5.9683 |
|  |  | $q_{2}$ | 1102.9284 | 1099.3056 | 1096.8586 | 1095.0928 | 1093.8525 |
|  | C | $q_{1}\times10^{8}$ | 5.2619 | 5.2808 | 5.2952 | 5.3056 | 5.3129 |
|  |  | $q_{2}$ | 1439.0631 | 1433.6236 | 1429.4799 | 1426.3667 | 1424.1450 |
|  | D | $q_{1}\times10^{8}$ | 4.0814 | 4.1195 | 4.1485 | 4.1717 | 4.1915 |
|  |  | $q_{2}$ | 1801.3347 | 1791.9305 | 1783.8970 | 1777.6128 | 1773.0778 |
| 30 | A | $q_{1}\times10^{8}$ | 6.3487 | 6.3552 | 6.3595 | 6.3622 | 6.3638 |
|  |  | $q_{2}$ | 1081.2670 | 1080.7085 | 1080.3732 | 1080.1769 | 1080.0841 |
|  | B | $q_{1}\times10^{8}$ | 5.5896 | 5.5947 | 5.5976 | 5.5990 | 5.5997 |
|  |  | $q_{2}$ | 1359.5822 | 1356.4257 | 1354.3043 | 1352.9016 | 1351.9444 |
|  | C | $q_{1}\times10^{8}$ | 4.5368 | 4.5537 | 4.5650 | 4.5723 | 4.5771 |
|  |  | $q_{2}$ | 1585.5717 | 1580.1052 | 1576.5044 | 1574.0176 | 1572.4634 |
|  | D | $q_{1}\times10^{8}$ | 3.3224 | 3.3601 | 3.3872 | 3.4075 | 3.4216 |
|  |  | $q_{2}$ | 1975.7617 | 1964.2228 | 1955.6185 | 1949.2609 | 1944.2793 |
